# Sensory and Chemical Drivers of Wine Consumers’ Preference for a New Shiraz Wine Product Containing *Ganoderma* *lucidum* Extract as a Novel Ingredient

**DOI:** 10.3390/foods9020224

**Published:** 2020-02-20

**Authors:** Anh N.H. Nguyen, Trent E. Johnson, David W. Jeffery, Dimitra L. Capone, Lukas Danner, Susan E.P. Bastian

**Affiliations:** 1 Department of Wine and Food Science, School of Agriculture, Food and Wine, The University of Adelaide, 5064 Glen Osmond, Australiatrent.johnson@adelaide.edu.au (T.E.J.); david.jeffery@adelaide.edu.au (D.W.J.); dimitra.capone@adelaide.edu.au (D.L.C.); lukas.danner@adelaide.edu.au (L.D.); 2 Australian Research Council Training Centre for Innovative Wine Production, The University of Adelaide, 5064 Glen Osmond, Australia

**Keywords:** hedonic clusters, rate-all-that-apply (RATA), medicinal mushroom, wine volatile chemistry

## Abstract

This study explored wine consumers’ preferences towards a novel Australian Shiraz wine product containing *Ganoderma lucidum* (*GL*). Wine consumers (*n* = 124) were asked to complete a questionnaire and participate in a blind tasting of six *GL* wine products (differing in the amount and timing of *GL* extract additions). Based on individual liking scores for each *GL* wine product that was tasted, four hedonic clusters C1 (*n* = 44, preferred control and low levels of *GL* additions), C2 (*n* = 28, preferred control only), C3 (*n* = 26, generally preferred all *GL* additions) and C4 (*n* = 26, preferred 1 g/L additions and 4 g/L post-fermentation) were identified. Sensory attributes of the *GL* wine products were also profiled with rate-all-that-apply (*n* = 65) and the 31 sensory attributes that significantly differentiated the wines underwent principal component analysis with the hedonic clusters overlaid to explain consumers’ preferences. There was a clear separation between hedonic clusters. Sensory attributes and volatile flavor compounds that significantly differentiated the wines were subjected to partial least squares regression, which indicated the important positive drivers of liking among the hedonic clusters. Pepper and jammy aroma, 3-methylbutanoic acid (linked to fruity notes) and non-fruit aftertaste positively drove C2′s preference, whereas spice flavor and hexanoic acid (known for leafy and woody descriptors) drove C3′s liking. There were no positive drivers for C1′s liking but bitter taste, cooked vegetable, and toasty aromas drove this cluster’ dislike. C4 preferred brown appearance, tobacco aroma, and jammy and cooked vegetable flavors. These findings provide the wine industry with deeper insights into consumers’ liking towards new *GL* wine products targeted at the Australasian market.

## 1. Introduction

*Ganoderma lucidum* (*GL*), a highly revered curative mushroom, has been commonly used for more than 2000 years due to its promotion of longevity and prevention of diseases [1,2,3,4,5], which have drawn the attention of researchers. Furthermore, both researchers and consumers from different countries, including some Asian countries, North America, and Europe, are interested in the application of *GL* in the production of health-supporting products [6]. For example, in China, the presence of *GL* extract during the fermentation process of soy milk improved the health benefits as well as the consumer acceptability of the product [7]. Other products, such as Chinese *GL Lycium chinensis* Miller tomato wine [8] and yogurt [9,10] with *GL* extract added during production, have also been described. Even alcoholic beverages have been investigated; Serbian Pilsner beer and brandy had *GL* added aseptically to create a better-perceived body in the beer [11] and a brandy [12] with functional properties. Recently, Ghobadi, et al. [13] demonstrated that *GL* powder is an effective preservative in the production of sausage or in the meat industry. However, to the best of our knowledge, there is currently no study on the development of a novel *GL* grape wine product for the Australian and potential global wine markets.

There is no doubt that every consumer has a unique pattern of taste preferences, resulting in consumers displaying a diverse range of behaviors [14] that can be difficult to interpret but could be related to differences in socio-demographics such as individual knowledge, cultural background, age, gender, etc. Market segmentation is a technique identifying consumers within a specific market who have similar wants, needs, and behaviors [15]. Meilgaard, et al. [16] suggested that consumers with similar attitudes might be identified by cluster analysis. Many segmentation bases have been applied in the literature, with examples for wine consumers that include liking scores [17,18,19], knowledge, or product-related experience [20,21], demographics [22] and psychographic segmentation bases such as personality, values or interest. Besides the application of geographic, demographic and socio-economic, behavioral, and psychographic segmentation bases in the wine market, other segmentation bases in various wine market models are emerging, including those based on biology, sustainability and social media [23]. Notably, consumer acceptance and behavior studies appear to favor hedonic clustering whereas marketing-focused research is more aligned with the other criteria such as geographic, demographic, and so on. However, the combination of these two distinct bases can provide powerful additional insights into consumer responses [14,24].

To date, consumers’ acceptance and attitudes towards a new wine product with *GL* extract in relation to liking, product knowledge, and demographic differences have not been well-documented in Australia. There is only one consumer study relating consumers’ attitudes toward a new Australian wine product containing *GL* reported by Nguyen, et al. [25], who revealed that Vietnamese consumers were more interested in new *GL* wine products compared to Australian and Chinese consumers. Indeed, despite the promise of wine supplemented with traditionally revered foods such as *GL*, no studies have focused on consumers’ acceptance of *GL* wine products, from the perspective of individual liking and demographic differences in conjunction with sensory and chemical profiling of these beverages. In addition, distinct from the famous flavored German vermouth and Greek retsina wines that still exist today, wine products commonly available on the market are usually flavored with botanicals including fruits or spices. A *GL* grape wine product on the other hand, is an innovative wine product concept as it contains functional ingredients common in traditional Asian medicine that may also possess distinctive sensory profiles appealing to current traditional and emerging wine consumer segments. Therefore, there was a need to create and investigate consumer liking of *GL* wine product prototypes.

To profile products’ sensory characteristics, numerous studies have proposed several sensory methods that are suitable for naïve consumers such as napping, free choice profiling, flash profiling, check-all-that-apply (CATA), and rate-all-that-apply (RATA) [26,27,28]. According to Jaeger and Ares [29] and Danner, Crump, Croker, Gambetta, Johnson, and Bastian [28], RATA is an economical and flexible rapid method suitable to reliably profile a variety of products using naïve consumers as subjects. Our previous study generated sensory and volatile flavor profiles of *GL* wine products [30]. Lower levels of extract produced smooth wines with red fruit, floral and confectionery notes, while wines with higher levels of extract were more complex with woody, dried fruit, earthy, and mushroom notes and bitter taste. The latter is likely due to the bitterness derived from the triterpenes in the *GL* extract [30]. As the results indicated that the presence of differing levels of *GL* extract produced wine products with distinctive attributes, these may suit varying consumer tastes. This study aimed to investigate the sensory attributes and chemical composition driving consumer clusters’ liking of *GL* wine products using a consumer-centric, new product development approach. A combination of a rapid sensory method using naïve consumers (RATA), basic analytical chemistry, and volatile chemical analysis using headspace-solid-phase micro-extraction (HS-SPME) with gas chromatography-mass spectrometry (GC-MS), followed by consumer acceptance testing, and hedonic segmentation, was employed.

Responding to these gaps in current knowledge, this study aimed to investigate:

- Consumers’ acceptance of novel *GL* wine products.
- The most preferred level of *GL* extract added prior to or post-fermentation in the production of these novel wines.
- Consumer profiles of *GL* wine hedonic clusters.
- The sensory attributes in *GL* wine products important for driving consumer hedonic cluster responses.
- The identification of the sensory and chemical parameters most important to consumer *GL* wine products liking using prediction models.

This knowledge might assist to increase the competitiveness of the Australian wine industry locally and in its many wine export countries.

## 2. Materials and Methods

### 2.1. Samples

Six Australian Shiraz wines were used for a blind consumer RATA sensory panel and underwent chemical composition analyses. These were selected from 18 wines (six wines produced in triplicate), made with or without *GL* extract added at different stages of the fermentation process described in Nguyen, et al. [30]. Based on the Australian and New Zealand Food Standards (Standard 2.7.4), such wines containing *GL* would be considered a “wine product” but will be referred to as wine throughout the remainder of the text for simplicity.

The samples included wines fermented with three different levels of *GL* extracts added prior to inoculation of yeast for primary fermentation, namely 1 g/L (PRE 1), 2 g/L (PRE 2) and 4 g/L (PRE 4); wines enriched with two levels of *GL* after fermentation, being 1 g/L (POST 1) and 4 g/L (POST 4), and a wine without addition of *GL* (control). All wine samples were stored at 15 °C and acclimatized to room temperature (22–23 °C) a day before serving.

### 2.2. Chemical and Sensory Measurements

#### 2.2.1. Basic Wine Composition

Basic chemical parameters of the wine samples were reported in a previous study [30] using methods described in Iland, et al. [31]. Basic measurements included pH and titratable acidity (TA), volatile acidity (VA), free and total SO_2_ content, wine color (CIELAB), ethanol content (% *v*/*v*) and residual sugars. VA was measured in duplicate whereas other measurements were conducted in triplicate.

#### 2.2.2. HS-SPME GC-MS Analysis of Wines

HS-SPME coupled with GC-MS was used to identify and quantify key volatiles in *GL* wines. Twenty-nine volatile compounds reported in a previous study [30] were used for PLS analysis in conjunction with sensory data from the current study.

#### 2.2.3. Rate-All-That-Apply (RATA) Sensory Panel

The Shiraz wines were assessed by 65 participants who were aged 28 to 35 years (51% female) and had consumed wine in the last 12 months. The sensory panel used RATA with a seven-point intensity scale, anchored from 1 = “extremely low” to 7 = “extremely high”. RATA evaluations were conducted as described in Danner, Crump, Croker, Gambetta, Johnson, and Bastian [28].

### 2.3. Consumer Sample

Wine consumers (*n* = 124, 52% female) were recruited at a food market in Adelaide, South Australia, where the service of alcohol to consumers was permitted through a limited alcohol license obtained by the researchers. Inclusion criteria required respondents to be of legal drinking age (i.e., above 18 years old) and to have consumed grape wine within the past 12 months. This study was approved by the Human Research Ethics Committee of The University of Adelaide (Approval No. H-2016-194).

### 2.4. Questionnaire

Survey Monkey^TM^ (Palo Alto, CA, USA; http://www.surveymonkey.com) was used for the consumer questionnaire, which consisted of five sections that covered demographic information, alcohol consumption behavior, wine knowledge, consumer behavior towards *GL* wines and a blind tasting of the *GL* wines. Section one—demographic questions—related to age, gender, education, household income, the preferred price for a bottle of wine, wine-drinking frequency, and the preferred place to purchase wine. The second section asked about wine purchase drivers with questions covering 14 factors used in choosing wines, where 1 = extremely unimportant, 5 = neither important nor unimportant and 9 = extremely important. Section three included questions that asked respondents to rate their liking score for the six *GL* wine samples in the blind tasting. In the last section, participants were asked to rate their level of agreement on a series of statements with respect to their attitudes towards novel *GL* wines. As with the previous study [25], the questionnaire was generated in English and underwent a pilot test by a small group of staff (*n* = 4) from the School of Agriculture, Food and Wine at the University of Adelaide, to clarify any ambiguity before being used for the consumer trial.

The consumer tasting sessions were designed using RedJade^®^ software (Redwood City, CA, USA) to produce a packing slip with an identifier number for each person and to determine the presentation order of the six wines. Wines (30 mL) were served at room temperature on an individual white tray for each person in randomized order, in transparent 215 mL International Organization for Standardization wine glasses labelled with three-digit codes and covered with glass lids. Participants were required to read the participant information sheet and consent to participate in the study before tasting the wines. Respondents were instructed to score their liking for each wine on a nine-point Likert category scale anchored by 1 = extremely dislike, 5 = neither like nor dislike, and 9 = extremely like. To cleanse the palate, participants were offered unsalted crackers and water plus were required to have a one-minute break between samples. Participants could taste the same sample again if necessary but could not go back and re-taste the previous sample.

### 2.5. Data Analyses

The data were analyzed using XLSTAT (Ver. 2017, Addinsoft, Paris, France). Basic chemical data were analyzed by one-way analysis of variance (ANOVA) with Tukey’s HSD, post hoc test. RATA data were analyzed by a mixed model ANOVA with assessors as random factors and Fisher’s LSD post hoc test for multiple comparisons. Thirty-one significant sensory attribute means and hedonic clusters as supplementary data were subjected to principal component analysis (PCA), and a biplot of cluster data and sensory attributes generated. For hedonic clustering, liking scores were subjected to a k-means cluster analysis. Chi-square tests with z-test for post hoc comparisons were used to test for differences in demographics between the four identified hedonic clusters. Differences in mean overall liking and liking between the four clusters were determined with a one-way ANOVA using LSD post hoc tests. Volatiles were also analyzed by one-way ANOVA with LSD post hoc test. Finally, significantly different volatile chemical components and sensory attributes were used as the predictor X-variables and the cluster mean liking scores as the Y-variables in partial least squares (PLS) regression analysis. The variables considered important for the prediction model were chosen based on variable importance in the projection VIP values > 1 [24]. All the statistical tests were conducted using a significance level of 0.05.

## 3. Results and Discussion

In our previous study, *GL* wines were made for the first time [30]. However, before potential release to the market, there was a need to examine consumers’ preferences in more detail and how they related to the wines’ sensory and chemical profiles. This may provide Australian winemakers with initial insights into the formulation of a novel wine product that might appeal to various markets.

### 3.1. Consumer Preference

#### 3.1.1. Overall Liking

Liking scores for *GL* wines are presented in Table 1 showing the liking ratings ranged from 4.1 to 5.2. These values are in accordance with previous studies with Australian consumers reporting mean liking ratings between 5 and 6 for high-quality commercial wine samples during a blind tasting [32,33]. This indicates that Australian consumers might be cautious when scoring wines tasted blind, and higher ratings might only be observed when additional information about the wines is presented together with the samples [33].

**Figure 1:**
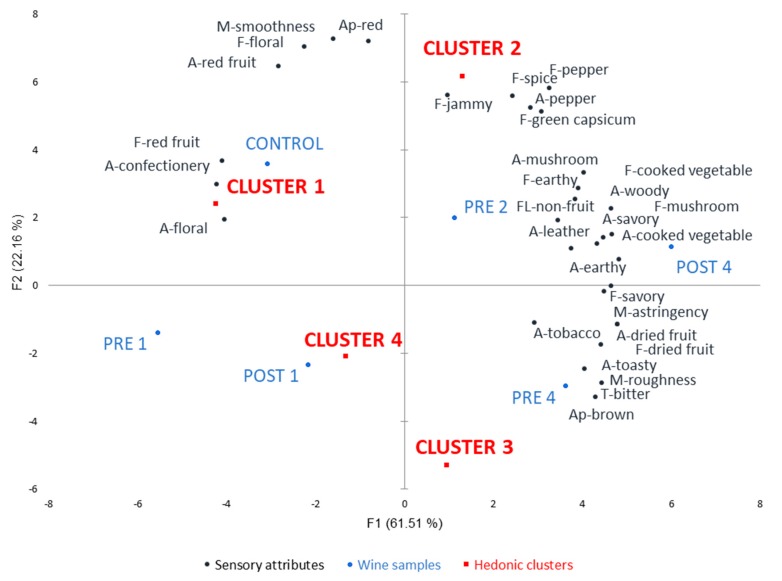
Principal component analysis (PCA) of 31 sensory attributes perceived significantly different between six *GL* wines and four distinct hedonic consumer clusters as supplementary data, including Cluster 1 (*n* = 44); Cluster 2 (*n* = 28); Cluster 3 (*n* = 26) and Cluster 4 (*n* = 26). Prefixes: A- = Aroma attribute; T- = taste; F- = flavor attribute; M- = mouthfeel, Ap- = appearance, FL- = aftertaste (fruit and non-fruit) intensity of different wine treatments, PRE = *GL* extracts added prior to fermentation (PRE 1, PRE 2 and PRE 4), and POST = *GL* extracts added after fermentation process (POST 1 and POST 4).

The mean hedonic data revealed statistical differences in the liking of both Control and the POST 1 g/L *GL* wine compared with that of the wines with higher *GL* additions (2 and 4 g/L) (Table 1). This was in line with a previous study conducted by Kim, et al. [34] who, using 50 trained panelists, demonstrated that Korean rice wine, Yakju, with *GL* added at 1 g/L was the most acceptable, but wines with higher levels of *GL* extracts resulted in an unfavorable taste due to the enhanced bitterness. Indeed, the Shiraz wines with higher amounts of *GL* extract (4 g/L) added pre- or post-fermentation were perceived as more bitter (Figure 1). In contrast, Pecic, et al. [35] reported that commercial grape brandy with 25 g/L *GL* addition had the best acceptability following sensory evaluation by five qualified experts. It is possible that unlike brandy, bitter wine compounds such as flavan-3-ols [36] present in the wines of the current study may have interacted with the relatively low levels of *GL* and had an additive effect on perceived bitter taste. Further investigation of the red wine polyphenolic and *GL* bitter compound content of *GL* red wines, and estimation of their rejection thresholds in red wine matrices is required. This may provide useful information for future product development projects in terms of the determination of *GL* levels that best suit consumers’ acceptability towards various kinds of *GL* foods and beverages. The wines in the current study were also made from red juice and not fermented with skins and seeds as is usually the case for dry red table wines. Fermentation of *GL* wines on skins would likely produce more full-flavored wines with more intense color and higher tannin [37], which would also modulate the impact of *GL* additions and potentially enhance consumer acceptability [38].

#### 3.1.2. Cluster Analysis of Consumers’ Hedonic Scores

In order to identify consumer clusters with different preferences for each of the *GL* wines, consumers were asked to report their liking for each *GL* wine on a nine-point hedonic category scale in the blind tasting. A recent review of studies segmenting the Australian domestic wine market revealed between three and five segments, depending on the type of segmentation base and respondent recruitment [39]. Using this knowledge, k-means cluster analyses were undertaken to produce 3, 4 and 5 cluster solutions. For segments to be viable, they must have the following characteristics: measurability; accessibility; substantiality and actionability [40].

When examining the 3 and 5 cluster solutions, it was found that some of the resultant clusters would fail one or more of the tests mentioned above. Additionally, we implemented the “elbow” method corresponding to the within-cluster sums of squares to assist with selection of the optimal number of clusters. In this instance, the inflection point on the line curve indicated that the underlying model fitted best at that point where K = 4. This four-cluster solution met the Kotler and Keller [40] criteria and was therefore preferred. The resultant clusters were: cluster 1 (C1, *n* = 44, 35.5%), cluster 2 (C2, *n* = 28, 22.5%), cluster 3 (C3, *n* = 26, 21.0%) and cluster 4 (C4, *n* = 26, 21.0%). C1 preferred the control and wines made with low levels of *GL* addition (control, POST 1, PRE 1 and PRE 2, mean liking scores of 5.9, 5.9, 5.8 and 5.1 respectively) (Table 1). C2 consisted of respondents who only liked the control wine. C3 liked all wines with *GL* additions, except for 1 g/L added post-fermentation (POST 1). C4 liked both wines with 1 g/L addition (PRE 1 and POST 1), plus the wine with 4 g/L added post-fermentation (POST 4). Knowing the influence that polymorphisms of genes responsible for bitter taste have on consumer food choice and preference [41], it would be of interest to examine the consumer cluster’s bitter plus other orosensory phenotypes and genotypes to see if this impacted cluster *GL* wine preferences. Furthermore, in the future, analyses of the wine polyphenolic profile and the *GL* extract bitter compound composition could provide more cluster liking insights. Perceived bitterness from *GL* addition may be moderated by retaining small amounts of residual sugars at the end of primary fermentation or adding grape juice concentrate post-ferment [37], which would potentially render the wines more palatable to more consumers.

#### 3.1.3. Demographics of Four Hedonic Clusters

Table 2 illustrates that there were some significant demographic differences between hedonic clusters in respect to non-tertiary education, household income under AU$100,000 and wine consumption frequency per month. When asked to express their agreement with various statements with respect to *GL* wines after the blind tasting, some significant differences were found in the responses between the clusters. The differing responses mostly related to C2 having a less positive opinion about *GL* wines and covered issues such as pricing of *GL* wine products; the acceptability of such products; whether there were health benefits associated with the wine product; and whether the addition of *GL* would impact their purchase decision (Table 3).

### 3.2. The Relationship between Hedonic Clusters and Sensory Characteristics of GL Wines

Moussaoui and Varela [26] concluded that RATA—an economical and rapid sensory profiling technique—was a suitable profiling method to use employing naïve consumers. This consumer RATA panel identified 31 of 54 attributes (57%) (*p* < 0.05) as significantly differentiating the *GL* wines (Supplementary Table S1). PCA was performed on the mean intensity ratings of the 31 significant attributes, with the hedonic clusters overlaid as supplementary data (Figure 1). The first two principal components (PCs) explained 83.7% of the variation in the data (PC1 = 61.51% and PC2 = 22.16) and revealed the relationships between the significant sensory attributes and each of the four hedonic clusters.

PC1 separated wines (PRE 4 and POST 4) that had a brown appearance, dried fruit and savory notes, earthy, leather, toasty and woody aromas, mushroom flavor, bitter taste, and astringent mouthfeel from those with confectionery and floral aromas and red fruit flavor (control, PRE 1 and POST 1). Wines characterized by brown appearance, tobacco aroma, and rough mouthfeel were differentiated along PC2 from those that were red in appearance, had red fruit aroma and floral flavor, and a smooth mouthfeel.

A clear separation was observed between hedonic clusters along both PC1 and PC2, with different sensory attributes driving hedonic scores for each cluster (Figure 1). C1 preferred wines with 1 g/L *GL* added and without *GL* (control, PRE 1, POST 1 and PRE 2) and appeared especially accepting of floral and confectionery aromas, red fruit note along with a smooth mouthfeel. C2 significantly preferred wine without *GL* (control) with more red appearance, red fruit aroma, floral flavor, and smooth mouthfeel. This is interesting, as this cluster was also less keen to drink wines with *GL*, thought *GL* wines were significantly less socially acceptable and were less likely to agree with the statement that these wines with health benefits were the way of the future (Table 3). To some extent, C2 also liked wine with 2 g/L *GL* and in particular jammy, spice, pepper, and green capsicum flavors. On the contrary, C3 preferred all the wines with low and high *GL* levels and in particular, PRE 4, which possessed a more brown appearance, dried fruit notes, toasty and tobacco aromas, a bitter taste and rougher mouthfeel. Lastly, C4 preferred wines made with both pre- and post-additions of 1 g/L *GL* but also attributes such as woody, leather, earthy, mushroom, cooked vegetable, savory and a more astringent mouthfeel in the wine with 4 g/L *GL* added post-fermentation (POST 4).

Considering the literature that has examined the sensory drivers and chemical composition of red wine consumer liking to date, there seem to be patterns emerging: i.e., those segments that like simple, red fruit, confected and smooth wines such as C1 and C2 in the current study; those who prefer wines with oak or oak-like notes, for example, the tobacco and woody notes liked by C3 and C4 in the current study; possibly another group who, like C3 and C4, also prefer more complex wines, with some texture plus spice, green notes, and savory nuances, namely C2 as evident in the present work [24,42,43]. Intriguingly, C3 members were partial to wines rated as relatively more bitter in taste. There are increasing reports on inter-individual variation in food and beverage preference due to human taste phenotypes. It would be interesting to determine whether this segment contained non-tasters with respect to PROP or PTC compounds [44]. Using different *GL* concentrations in the winemaking process and also involving different grape varietals, oak fermentation or maturation, and varying cap management and fining treatments could develop a wider range of wines with new profiles characterized by the sensory attributes identified above, that potentially drive individual hedonic responses by consumers. 

### 3.3. Sensory and Chemical Drivers of Liking

**Figure 2:**
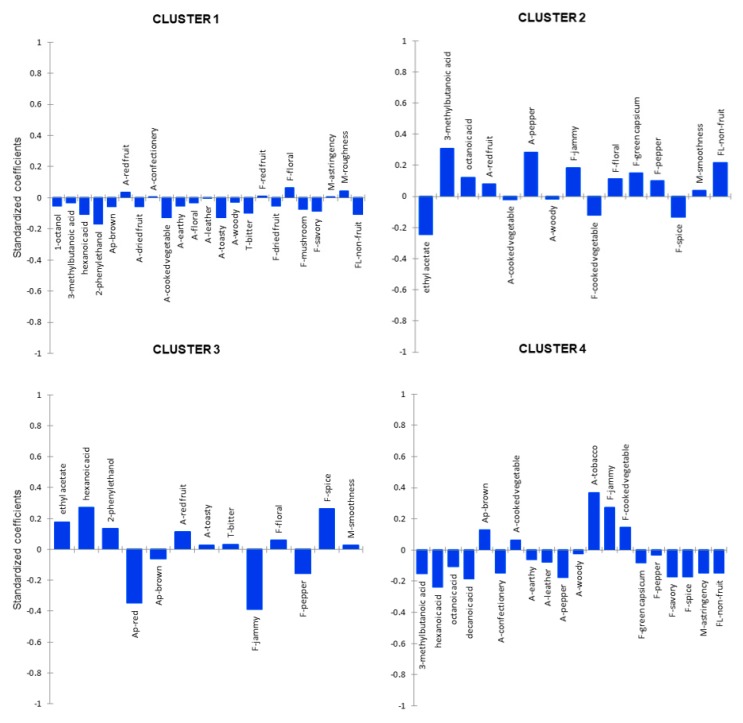
Standardized coefficients of the partial least squares (PLS) regressions by hedonic cluster using liking scores as Y-variables, and sensory attributes and volatiles as X-variables. Prefixes: A- = Aroma attribute; T- = taste; F- = flavor attribute; M- = mouth-feel, Ap- = appearance, FL- = aftertaste (fruit and non-fruit) intensity of different wine treatments.

It is widely accepted that for commercial success, it is advantageous for wine producers to understand the sensory attributes that influence consumer preference for their wines. This is particularly pertinent in the context of the current study, as the prototype Australian Shiraz *GL* wines produced represented a novel concept. As such, it was not clear what the wines made with *GL* extracts would smell, taste and feel like, and the potential drivers of consumer liking or disliking of these novel wines were also unknown. Basic chemical composition parameters (Supplementary Table S2) were analyzed and found to significantly differentiate treatments, but as the differences between treatments were below perceived difference thresholds, they were not included in the final PLS analysis. Thus, 31 sensory attributes and eight volatile compounds (2-phenylethanol, 1-octanol, ethyl acetate, limonene, and hexanoic, octanoic, decanoic and 3-methylbutanoic acids) that significantly differentiated the wine treatments were subjected to PLS regression. The resultant regression standardized coefficients for the four clusters are shown in Figure 2.

Drivers are considered the most important when the standardized regression coefficient absolute values are higher than 0.1 [24]. Figure 2 showed that most of the potential drivers including sensory attributes and volatile compounds did not predominantly influence the overall liking across the four hedonic clusters (regression coefficients < 0.1). The majority of drivers of C1 liking were related to negative coefficients, with the important negative drivers being 2-phenylethanol (contributing to floral and rose attributes [30]), toasty and cooked vegetable aromas, bitter taste and non-fruit flavor aftertaste (Figure 2) as the regression coefficients were < -0.1. This can probably explain why C1 preferred the control wines and those with 1 g/L *GL* added, not having floral or rose (2-phenylethanol), or toasty, cooked vegetable aromas. Ethyl acetate and cooked vegetable and spice flavors were negative drivers of C2′s liking, while positive liking drivers of C2 were 3-methylbutanoic acid (banana, pear), octanoic acid (typically fruity attributes) [30] and pepper aroma, jammy, floral, and green capsicum flavors plus non-fruit flavor aftertaste, which provides partial explanation for this cluster’s preference for the fruity control and peppery and more textural PRE 2 wines. Red appearance together with pepper and jammy flavors negatively drove C3′s liking, while ethyl acetate, hexanoic acid, 2-phenylethanol, and red fruit aroma and spice flavor positively drove preference of this cluster. The positive drivers of C4′s liking were brown appearance, tobacco aromas and jammy and cooked vegetable flavors, probably explaining their preference for the POST 4 wine. Negative drivers included 3-methylbutanoic acid, hexanoic acid, octanoic acid, decanoic acid, confectionery and pepper aromas, savory and spice flavors and astringent and non-fruit length; all characters not as apparent in the PRE and POST 1 wines.

Identification of four consumer clusters was achieved, each with different positive and negative drivers of preference. When predicting the preference of the total consumer group, none of the variables strongly drove consumers’ disliking (data not shown). This analysis supports the potential for hedonic clustering techniques, married with other sensory and chemical data, to provide powerful consumer information that can be used in either developing new wine products, or better targeting consumer segments.

## 4. Study Limitations

The present study findings are subject to some limitations. Even though consumers were randomly invited to taste the wines at the market place, those that participated in the study were likely more interested in and involved with wine than regular consumers, which could lead to a higher level of wine knowledge as well as hedonic ratings. As only a limited number of volatile and sensory attributes were predicting cluster liking, it is possible other wine product components such as the polyphenols or *GL* extract compounds not measured in the current study could provide further explanations for consumer preference.

## 5. Conclusions

Key barriers to the rapid success of Australian wines in Australasian markets are the lack of understanding of wine consumers’ taste preferences and possibly the lack of involvement of consumers in the co-creation process of the wine. We aimed to investigate the development of a wine prototype for a specific target market, which evolved from the solicited consumer attitudes for *GL*-based wine presented in our previous work. Extending on that, RATA was used to assess consumer perceptions of Shiraz-based *GL* wine products, and the resulting sensory results in conjunction with wine composition were linked to the preference of consumer segments.

Although there were differences in hedonic scores for each *GL* wine corresponding to its sensory characteristics, most of the responses showed that consumers were very likely to be interested in the new Australian *GL* wines. The results of the study suggested that *GL* wines containing a low amount (i.e., 1 g/L) of *GL* extract, added either pre- or post-fermentation, displayed broader preference agreement and may form the basis for new wine products aimed at the market as a whole. Yet, this work also identified consumer segments that liked wines with higher *GL* additions, namely C3 and C4. This suggests that there is scope to make more individualized products for these specific targets. Examination of other secondary metabolites in *GL* wines derived from either the extract or grape will help explain sensory attributes and consumer preferences further. Leading on from this, deeper understanding of the basis of the inter-individual variation amongst consumers could be a future area of investigation, for example, profiling the consumers’ phenotypic or genotypic profiles for personalized targeting of products. In addition, consumer demographics, wine consumption behavior, and attitudes towards *GL* wines of each hedonic cluster were statistically described and some nuanced differences identified.

Better understanding of the chemical and sensory drivers of consumers’ perception and preference for new *GL* wines, and incorporating consumers in the development process (i.e., co-creation), provided a robust approach that others could adopt to obtain deeper insights into the needs of a target market for novel products. These findings may guide producers wishing to make *GL* wine products to incorporate production processes to promote or demote specific characters to meet specific consumers’ needs. The study was the first to explore consumers’ preference towards novel wines with different levels of *GL* additions. Future research is recommended to investigate how different production methods (such as winemaking with skin contact in the fermentation or with different grape varieties, oak contact, and aging) would impact sensory and chemical profiles of the *GL* wines for the purposes of an additional range of wine products for potential markets of the *GL* wine product category.

## Figures and Tables

**Table 1:** Consumers’ overall liking means for the whole cohort and by consumer clusters of six *Ganoderma lucidum (GL)* wines.

Wine Samples	Overall Liking	Cluster 1 (*n* = 44)	Cluster 2 (*n* = 28)	Cluster 3 (*n* = 26)	Cluster 4 (*n* = 26)
Control	5.2 a (1.82)	5.9 a	6.0 a	4.0 c	4.6 bc
POST 1	5.2 a (2.12)	5.9 a	3.8 cd	4.6 bc	6.2 a
PRE 1	5.1 ab (1.96)	5.8 a	3.1 d	5.9 a	5.4 ab
PRE 2	4.6 bc (2.10)	5.1 b	5.0 ab	5.1 ab	3.1 b
POST 4	4.5 cd (1.91)	3.7 c	4.6 bc	5.1 ab	5.5 ab
PRE 4	4.1 d (2.18)	2.9 d	4.5 bc	6.0 a	3.7 cd

Data were collected with a nine-point hedonic Likert category scale anchored by 1 = extremely dislike, 5 = neither like nor dislike, and 9 = extremely like and analyzed by one-way ANOVA, with Fisher’s LSD post hoc tests and a significance level of *p* < 0.05. Different letters within a column indicate significant differences between mean wine liking scores. Standard deviation in parentheses.

**Table 2:** Demographics of the four hedonic clusters.

	Total (*n* = 124)	Cluster 1 (*n* = 44)	Cluster 2 (*n* = 28)	Cluster 3 (*n* = 26)	Cluster 4 (*n* = 26)
**Gender**					
Male	47.6	45.5	46.4	50.0	50.0
Female	52.4	54.5	53.6	50.0	50.0
**Age**					
18–34	41.9	43.2	39.3	42.3	42.3
35–54	33.1	34.1	25.0	38.5	34.6
+55	25.0	22.7	35.7	19.2	23.1
**Education**					
Non-tertiary	42.7	45.5 a	42.9 ab	26.9 b	53.8 a
Bachelor’s degree	29.0	29.5	32.1	38.5	15.4
Post-graduate degree	28.3	25.0	25.0	34.6	30.8
**Household income (AU$)**					
<$50,000	52.4	63.6 a	39.3 b	53.8 ab	46.2 ab
$50,001–$100,000	29.8	18.2 a	42.9 b	26.9 ab	38.5 ab
$100,001–$200,000	12.9	18.2	10.7	15.4	3.8
>$200,000	4.9	0.0	7.1	3.8	11.5
**Price per 750 mL bottle of wine (AU$)**					
less than $15	22.6	27.3	17.9	11.5	30.8
$15–$29	53.2	45.5	60.7	57.7	53.8
$30–$49	19.4	20.5	21.4	23.1	11.5
$50–$100	1.6	0.0	0.0	3.8	3.8
More than $100	1.6	2.3	0.0	3.8	0.0
Never purchase	1.6	4.5	0.0	0.0	0.0
**Wine consumption frequency**					
Few times per week	50.0	45.5	53.6	65.4	38.5
Once per week	16.9	20.5	17.9	11.5	15.4
Once per two weeks	13.7	9.1	17.9	15.4	15.4
Once per month	19.4	25.0 ab	10.7 ab	7.7 b	30.8 a
**Place of wine purchase**					
Online wine/liquor store	8.9	4.5	17.9	7.7	7.7
Wineries/cell door	12.9	13.6	7.1	19.2	11.5
Retail chain liquor store	66.1	68.2	64.3	61.5	69.2
Independent wine store	3.2	2.3	3.6	3.8	3.8
Restaurant	4.0	4.5	3.6	0.0	7.7
Others (clubs, bars, hotels)	4.9	6.8	3.6	7.7	0.0

Data presented are percentages. Chi-square values for wine hedonic clusters of the demographic data were: gender, *X*^2^ = 0.217, df = 3, *p* = 0.975; age, *X*^2^ = 2.639, df = 6, *p* = 0.853; education, *X*^2^ = 5.611, df = 6, *p* = 0.468, income, *X*^2^ = 14.231, df = 9, *p* = 0.114, preferred price for a 750 mL bottle of wine, *X*^2^ = 13.071, df = 15, *p* = 0.597; wine consumption frequency, *X*^2^ = 9.520, df = 9, *p* = 0.391; place to purchase wine, *X*^2^ = 9.601, df = 15, *p* = 0.844. Different letters within a row indicate significant differences between wine hedonic clusters based on z-test at significance level *p* < 0.05.

**Table 3:** Australian consumers’ attitudes towards *GL* wine statements and their market expectations.

	Cluster 1 (*n* = 44)		Cluster 2 (*n* = 28)		Cluster 3 (*n* = 26)		Cluster 4 (*n* = 26)		*p*-Value
	Mean	Std. Error	Mean	Std. Error	Mean	Std. Error	Mean	Std. Error	
I do not know about the *GL* wine but I think it is worth trying.	6.2	0.35	6.0	0.44	5.5	0.46	5.3	0.46	0.381
I would like to go to places where *GL* wines are served.	5.0	0.31	4.6	0.38	4.7	0.40	5.0	0.40	0.766
I would drink almost any *GL* wine.	4.4	0.32	3.8	0.40	4.2	0.41	4.5	0.41	0.537
At a social gathering, I will try *GL* wine.	6.6	0.28	7.1	0.35	6.4	0.37	6.2	0.37	0.278
I am keen on drinking *GL* wine if the price is reasonable	5.9 a	0.33	4.7 b	0.42	5.6 ab	0.43	5.8 ab	0.43	**0.020**
Not sound “romantic”.	5.1	0.34	5.6	0.42	5.3	0.44	4.5	0.44	0.362
Are not as socially acceptable or impressive.	4.1 ab	0.33	5.2 a	0.42	4.8 ab	0.43	4.0 b	0.43	**0.050**
Should have this information specified on the label.	6.4	0.31	7.3	0.39	6.5	0.40	6.8	0.40	0.344
Does not matter to me as long as *GL* wine tastes good.	5.3	0.36	5.8	0.46	5.8	0.47	4.9	0.47	0.402
Are the way of the future regarding health benefits	5.9 a	0.28	4.9 b	0.35	5.3 ab	0.37	5.6 ab	0.37	**0.030**
Have no influence on my purchase decision.	5.2 a	0.32	5.3 a	0.40	4.0 b	0.42	4.8 ab	0.42	**0.030**
Are cheap or of lower quality.	5.0	0.25	5.0	0.32	4.4	0.33	4.7	0.33	0.481

Data presented are mean agreement scores; where 1 = highly disagree, 5 = neither agree nor disagree and 9 = highly agree. Different letters within a row indicate significant differences between wine hedonic clusters, data analyzed by one-way ANOVA, Fisher’s LSD with significant level at *p* < 0.05 indicated in bold.

## Supplementary Materials

The following are available online at https://www.mdpi.com/2304-8158/9/2/224/s1, Table S1: Mean intensity ratings of sensory attributes that significantly differentiated six *GL* wines from RATA sensory testing, Table S2: Basic chemical composition of the *GL* wines.
