# Supplementary material for: Sensory and Chemical Drivers of Wine Consumers’ Preference for a New Shiraz Wine Product Containing Ganoderma lucidum Extract as a Novel Ingredient
Source: Foods. 2020 Feb 20;9(2):224. doi: 10.3390/foods9020224 (PMC7074515; doi:10.3390/foods9020224)
Supplement: Supplementary file 1 [file foods-09-00224-s001.pdf]

SUPPLEMENTARY INFORMATION FOR

Sensory and chemical drivers of wine consumers' preference for a new Shiraz wine product containing *Ganoderma lucidum* extract as a novel ingredient

Anh N.H. Nguyen [1], Trent E. Johnson [1], David W. Jeffery [1,2], Dimitra L. Capone [1,2], Lukas Danner [1], and Susan E.P. Bastian [1,2,*]

[1] Department of Wine and Food Science, School of Agriculture, Food and Wine, The University of Adelaide, PMB 1, Glen Osmond, South Australia, 5064, Australia.
[2] Australian Research Council Training Centre for Innovative Wine Production, The University of Adelaide, PMB 1, Glen Osmond, South Australia, 5064, Australia.
[*] Corresponding author sue.bastian@adelaide.edu.au; tel: +61 883136647

TABLE OF CONTENTS

	Page
**Table S1.** Mean intensity ratings of sensory attributes that significantly differentiated six *GL* wines from RATA sensory testing.	S2
**Table S2.** Basic chemical composition of the *GL* wines.	S3

Table S1. Mean intensity ratings of sensory attributes that significantly differentiated six *GL* wines from RATA sensory testing.

Sensory attributes	CONTROL	PRE 1	PRE 2	PRE 4	POST 1	POST 4
Ap-red	4.31 a	3.75 c	4.11 ab	3.72 c	3.91 bc	3.91 bc
Ap-brown	2.11 d	2.35 cd	2.60 bc	3.68 a	2.91 b	3.91 a
A-red fruit	3.86 a	3.45 ab	3.43 ab	2.57 c	2.95 bc	3.05 bc
A-dried fruit	1.55 c	1.54 c	1.98 abc	2.20 ab	1.75 bc	2.34 a
A-confectionery	2.83 a	2.89 a	2.65 ab	2.25 b	2.42 ab	2.15 b
A-cooked vegetable	0.57 ab	0.25 c	0.34 bc	0.78 a	0.20 c	0.80 a
A-earthy	0.77 bcd	0.62 d	1.05 abc	1.12 ab	0.75 cd	1.28 a
A-floral	2.32 ab	2.51 a	1.88 bc	1.75 c	1.94 bc	1.88 bc
A-mushroom	0.52 bc	0.42 c	0.78 ab	0.57 abc	0.51 bc	0.89 a
A-leather	0.49 bc	0.28 c	0.74 ab	0.71 ab	0.51 bc	0.85 a
A-pepper	0.74 a	0.31 b	0.68 a	0.65 a	0.34 b	0.66 a
A-savory	0.68 bc	0.54 c	1.09 b	0.97 b	0.82 bc	1.60 a
A-toasty	0.78 b	0.80 b	0.94 b	1.37 a	0.77 b	1.34 a
A-woody	0.85 bc	0.60 c	1.06 ab	1.03 ab	0.77 bc	1.28 a
A-tobacco	0.55 b	0.68 b	0.45 b	0.72 b	0.57 b	1.05 a
T-bitter	3.05 b	3.17 b	3.40 ab	3.77 a	3.25 b	3.69 a
F-red fruit	3.55 a	3.62 a	3.25 ab	2.77 c	3.06 bc	2.91 bc
F-dried fruit	1.32 c	1.29 bc	1.72 a	1.97 c	1.54 abc	2.15 ab
F-jammy	1.97 a	1.38 bc	1.78 abc	1.37 c	1.85 ab	1.98 a
F-cooked vegetable	0.46 ab	0.25 b	0.35 b	0.49 ab	0.22 b	0.71 a
F-earthy	0.68 bc	0.49 c	0.97 ab	0.71 bc	0.82 bc	1.15 a
F-floral	2.23 a	1.80 ab	2.00 ab	1.14 c	1.54 bc	1.65 b
F-mushroom	0.31 c	0.37 bc	0.65 ab	0.58 bc	0.29 c	0.92 a
F-green capsicum	0.72 a	0.38 b	0.63 ab	0.65 ab	0.35 b	0.72 a
F-pepper	0.82 abc	0.42 d	0.94 ab	0.63 bcd	0.55 cd	1.00 a
F-savory	0.71 b	0.65 b	1.17 a	1.29 a	0.69 b	1.32 a
F-spice	1.46 abc	1.34 bc	1.83 a	1.31 bc	1.08 c	1.75 ab
M-astringency	2.57 ab	2.35 b	2.85 a	2.91 a	2.68 ab	2.88 a
M-smoothness	3.75 a	3.40 ab	3.40 ab	3.03 b	3.18 b	3.43 ab
M-roughness	2.25 bc	2.14 c	2.60 ab	2.72 a	2.65 a	2.69 a
FL-non-fruit	3.72 ab	3.31 c	3.60 abc	3.89 a	3.34 bc	3.72 ab

Means within a row followed by different letters are significantly different. Data were collected and analyzed by using mixed model with Fisher's LSD post-hoc tests, with a significance level of $p < 0.05$. Prefixes: A- = aroma attribute; T- = taste; F- = flavor attribute; M- = mouth-feel, Ap- = appearance, FL- = aftertaste (fruit and non-fruit) intensity of different wine treatments. Prefixes PRE = *GL* extracts added prior to fermentation (PRE 1, PRE 2 and PRE 4), POST = *GL* extracts added after fermentation process (POST 1 and POST 4).

Table S2. Basic chemical composition of the *GL* wines

Treatment samples	pH	TA (g/L)	Ethanol (% v/v)	VA (g/L)	Free SO_2 (mg/L)	Total SO_2 (mg/L)	Color (AU)	Sugar (g/L)
CONTROL	3.91 cd	4.56 ab	12.75 c	0.25 c	48.53 c	123.73 b	10.54 f	0.61 f
PRE 1	3.90 d	4.21 d	12.3 d	0.25 c	51.73 b	117.33 c	11.67 e	0.74 e
PRE 2	3.92 c	4.29 cd	13.16 b	0.26 c	52.8 b	125.87 b	11.83 d	0.98 d
PRE 4	3.96 a	4.48 b	12.76 c	0.29 b	58.66 a	133.87 a	12.56 c	1.56 b
POST 1	3.94 b	4.63 a	13.83 a	0.34 a	36.26 d	90.67 e	12.59 b	1.32 c
POST 4	3.97 a	4.35 c	13.48 ab	0.3 b	36.26 d	96.53 d	14.62 a	2.96 a

Data are means of triplicate measurements, except VA measurements were conducted in duplicate. Means within a column followed by different letters are significantly different (one-way ANOVA, Tukey's HSD, post-hoc, $p < 0.05$). Abbreviation: Titratable acidity (TA), volatile acidity (VA).
